# When Limited Clinical Time With Patients Meets Unlimited Online Information

**DOI:** 10.2196/79031

**Published:** 2025-09-26

**Authors:** Ilona Fridman, Skyler B Johnson, Heather M Derry-Vick

**Affiliations:** 1 Center for Discovery and Innovation Hackensack Meridian Health Nutley, NJ United States; 2 Hackensack Meridian School of Medicine Nutley, NJ United States; 3 Lombardi Comprehensive Cancer Center Georgetown University Washington, DC United States; 4 Department of Radiation Oncology Huntsman Cancer Institute University of Utah Salt Lake City, UT United States

**Keywords:** health misinformation, health policy, patient safety, patient education, online health information, patient information needs, oncology

## Abstract

As patients with cancer increasingly seek guidance from online sources, the patient-clinician relationship is at risk of being displaced by fragmented, often unreliable information. One of the primary drivers of this trend is the insufficient time available for in-depth, relational consultation with health care providers (HCPs). We argue that the current clinical routine, constrained by documentation and administrative demands, fails to allow adequate time for supporting the informational, emotional, and relational needs of patients navigating complex decisions. This shortfall undermines HCPs’ ability to engage patients in shared decision-making and weakens the foundation of trust between patient and HCP. For some patients, this can result in selecting less-effective treatments or turning away from evidence-based care toward unproven online alternatives. While policy reforms to reduce administrative burdens and free up time for patient education and counseling are essential, they are slow to materialize, making immediate, actionable steps at the clinician level more urgent. We propose a set of practical, evidence-informed strategies that clinicians can adopt today to help meet patients’ informational and emotional needs, strengthen patient-HCP relationships, and ensure that patients’ health care decisions fit their preferences and are supported by scientific evidence.

## Introduction

**Figure 1:**
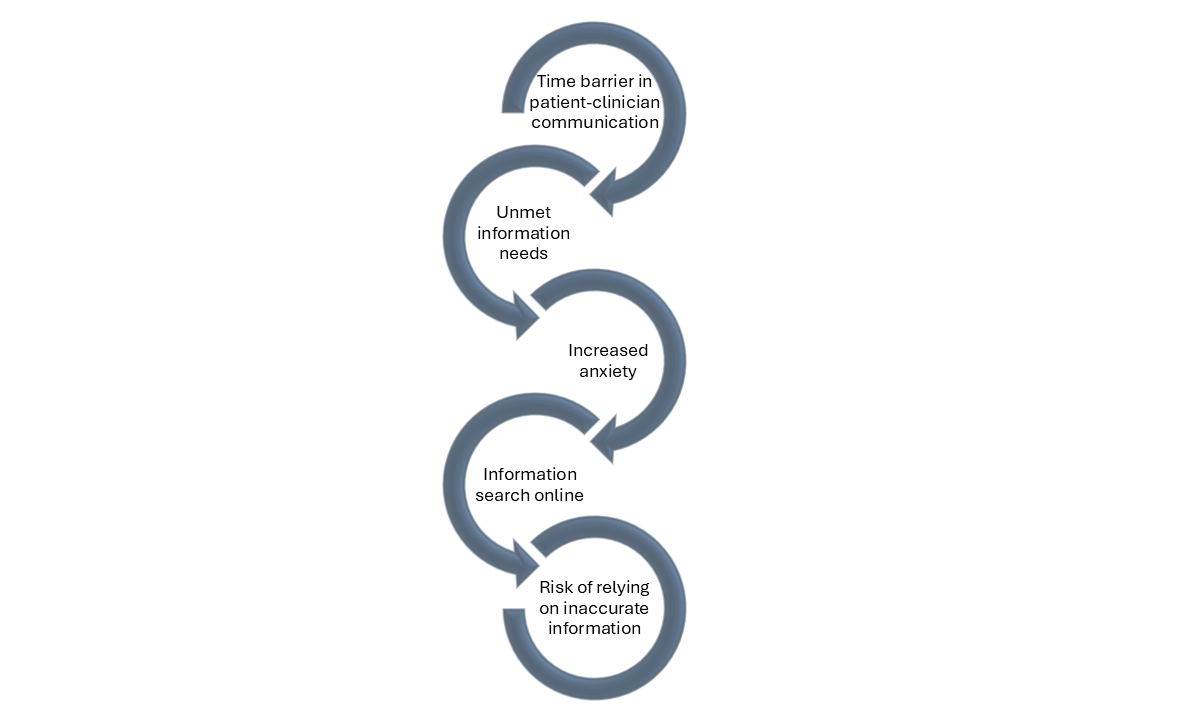
A pathway from limited clinical time for patient consultation to misinformed decisions by the patient.

One of the most pressing challenges in cancer care is insufficient time available for patient visits and patient-centered conversations [1]. While not the only factor, time constraints play a pivotal role in motivating patients with cancer to seek information from nonclinical sources, particularly those that they can find online [2-6]. Available at the fingertips of many patients and their caregivers, online cancer-related content is often either incomplete, unreliable, or dangerously inaccurate [7-10]. In this Viewpoint, focusing on the US health care system, we illustrate how limited time with health care providers (HCPs) contributes to patients’ reliance on nonclinical sources of information and increases their risk of making poorly informed, value-discordant decisions. Figure 1 shows a potential pathway to these decisions. While needed policy changes to rebalance HCPs’ time from administrative tasks and paperwork to patient counseling are yet to come, we identify and propose practical, actionable strategies to help HCPs mitigate the migration of patient trust to online sources.

## Time Barriers in Patient-Clinician Communication

A cancer diagnosis immerses patients into an emotionally charged and cognitively demanding landscape, where they must navigate complex clinical information, often under intense psychological stress. When people need to find cancer-related information, about 55% of them will reach a physician first, according to the Health Information National Trends Survey conducted in 2020 [11]. Patients place immense trust in their clinicians, seeking guidance in navigating complex treatment decisions. According to the National Trend Survey and Pew Research Center, more than half of patients believe they can consistently rely on their physicians to carry out care duties effectively, demonstrate care for patients’ interests, and offer accurate information [12,13]. However, when it comes to delivering cancer information to those who are looking for it, both patients and HCPs encounter a critical barrier—time availability for patient counseling.

Before 2012 [14], the average duration of an oncology outpatient encounter ranged from 17 to 23 minutes. While average visit length has remained relatively stable over time [15], the complexity of these visits has increased dramatically [16]. With the development of new treatments, the number of pages in practice guidelines for oncology has almost doubled over the same period [17]. Today, HCPs must not only assimilate a substantially greater volume of medical knowledge but also convey a share of that knowledge to patients in a clear and understandable manner within a similar time limit as 13 years ago. In addition to a dramatic increase in knowledge, systemic drivers play a role in the time pressures on clinicians. HCPs have to dedicate clinical time to managing referrals and orders, extensively documenting visits, seeking insurance preauthorizations, and navigating business-driven financial pressures to see more patients (eg, relative value unit–based reimbursement). These factors ultimately reduce HCPs’ availability for spending time with patients. Despite clinicians’ best efforts, the constraints of a typical clinical routine leave little room to fully address the range of informational needs regarding diagnoses, tests, and treatments, as well as physical and psychosocial burdens.

## Unmet Information Needs

Many newly diagnosed patients leave their medical appointments without a clear understanding of their treatment options [18], and they have unmet information needs related to supportive care, including information about psychological and physical adjustment to daily living and information about managing symptoms and side effects [19,20]. Although limited time is not the only factor contributing to patients’ unmet informational needs, it exacerbates other well-documented challenges, such as limited health literacy [21], delays in access to HCPs [22], and gaps in HCP communication skills [23]. Limited time with clinicians remains one of the key barriers to meeting patients’ information needs and exacerbates the other issues [24].

Time constraints make it difficult for patients to voice their preferences, ask clarifying questions, or request further explanation—all of which are essential to meaningful shared decision-making [25]. Limited time for shared decision-making is especially concerning, given that many cancer-directed treatments place a significant burden on patients, often involving life-altering side effects that patients must self-manage outside of clinical settings. If patients have unmet information needs or misunderstand their treatment options and potential consequences, they may be poorly prepared to adhere to their treatment, manage their side effects, and, thus, miss the opportunity to have a better quality of life and treatment outcomes [26-28]. Therefore, time for collaborative shared decision-making is critical not only for maintaining trusting relationships but also for optimal patient outcomes that are concordant with their goals.

## From Informational Needs to Anxiety

Unmet information needs increase uncertainty and, consequently, anxiety among patients with cancer. Patients who reported being unsatisfied with the information provided also reported higher levels of anxiety and depression, as well as a lower quality of life [29]. Further, among women with breast cancer, those with unmet information needs at baseline had increases in anxiety over time [30]. Notably, unmet informational needs that contribute to heightened anxiety can create a vicious cycle of negative experiences. Higher levels of anxiety correlate with patients’ ability to recall information from clinical encounters [31]. Furthermore, compared to patients with lower anxiety, those with higher anxiety report feeling less comfortable asking clinicians questions and trusting HCPs’ opinions [32]. In turn, clinicians may be less likely to initiate difficult conversations with anxious patients, leading to further disconnects in trusting patient-clinician relationships [33]. Although not the sole solution, allocating additional time to address patients’ informational needs is a critical component, especially during periods such as initial diagnosis or significant changes in disease status (eg, progression or recurrence). Investing time into informational needs is a time-efficient strategy: when patients’ needs are not met and collaborative, shared decision-making is not established, patients feel disconnected from their HCPs, and more time is required to rebuild trust.

## Information Search Online

As patient-centered communication weakens and uncertainty or anxiety builds, patients tend to invest more effort in seeking cancer information outside of clinical sources [2]. Although more than half of patients with cancer search for health-related information online, those who are dissatisfied with their care use online information significantly more [34]. In general, online information could be helpful and support patients’ education, preparing them for health-related conversations with their HCPs [35] and for self-management [36]. At the same time, patients use various sources of information, and the information provided is not always accurate. For instance, a recent study showed that up to 65% of people affected by cancer were willing to use social media to make medical decisions [37]. Another study indicated that 1 in 10 patients with cancer has based their medical decisions on the recommendations they identified on social media [38]. The quality of health information online, particularly on social media, remains questionable, with studies showing that up to 30% of posts related to cancer may be inaccurate, potentially leading patients toward harmful or suboptimal outcomes [7,39]. When evaluating the quality of online information, individuals tend to rely on observable factors, such as navigability, aesthetics, and ease of understanding, rather than on elements that truly reflect the information’s quality [40]. Thus, without proper consultation with health care experts, patients who search for information online are at risk of making misinformed decisions. The more patients rely on potentially unreliable sources, the more they become disconnected from their clinicians (ie, evidence-based sources), and the more time clinicians must spend later correcting misconceptions. Proactively addressing patients’ informational needs can help prevent misunderstandings and reduce the time required to correct patients’ knowledge in future visits.

## Risk of Misinformed Decisions

When patients cannot meet their informational needs or verify information with their trusted HCP, they face a risk of making suboptimal decisions that lead to poor clinical outcomes. For example, patients who use prescribed medication and rely on digital sources were 18% more likely to report low adherence than those who relied on health professionals (62% vs 44%) [41]. Similarly, use and trust in information from mass media and social media platforms undermined patient understanding of health-related information and reduced adherence to medical recommendations in different contexts [42,43]. In the cancer context, inaccurate and unreliable information often appears in the form of overly optimistic claims about complementary or alternative therapies. While some complementary therapies may offer great benefits for patients with cancer (particularly for managing symptoms) [44], if used without health care expert guidance, complementary medicine may lead to harmful outcomes. For instance, patients with cancer who use complementary medicine relying on inaccurate information tend to delay or report nonadherence to evidence-based treatments [45-47]. Furthermore, those patients who delayed evidence-based cancer-directed therapy and relied solely on complementary approaches had a 2-fold greater risk of death compared to those who did not delay standard therapy [48].

Time as a main barrier for discussing complementary medicine and other patient-identified information from nonclinical sources has long been recognized [49]. Yet, despite significant changes in the informational landscape in the last decade, where patients with cancer now have greater access to medical advice of varying quality through web sources and social media, this barrier remains unaddressed [50].

Importantly, dedicating more time to a discussion might not be enough for patients who are relying on nonclinical sources of information. Without proper training, patient-clinician communication may fall short. Currently, up to 37% of patients with cancer reported having negative experiences when bringing to HCPs information they found on their own [39]. Compared to those with positive experiences, these patients express significantly lower trust in information provided by HCPs [39]. This evidence suggests that discussion of patient-identified information likely requires not only a time commitment during a visit but also requires HCPs to invest time in receiving training for improving communication skills. Thus, supporting patients at the time of diagnosis and across the cancer continuum by addressing their informational needs, helping them access reliable sources, and validating their findings may not only lead to better outcomes but also help maintain trust and make clinical consultations more time-efficient and impactful.

## Action Plan for Insufficient Time With Patients

As patients increasingly turn to the internet and social media for answers to questions that could not be addressed in their visits—often at great personal cost—there is an urgent, unaddressed crisis at hand. As illustrated above, unmet informational and emotional needs frequently drive patients to seek support elsewhere and rely on nonclinical, potentially unreliable recommendations. While not the sole factor, limited time with HCPs remains a significant contributor to this concerning pattern. Increasing meaningful interactions and optimizing available time with the health care team can help patients better meet these needs and make informed, evidence-based decisions. Importantly, critical adjustments do not always require extensive effort. How can HCPs meet patient needs when more time is not an option? We summarize in Table 1 several strategies to optimize interactions with patients and provide examples from prior literature.

**Table 1:** Time-saving strategies for patient education in a digital information era.

Identified issue and recommendations		Implementation	Examples of using the recommended approach
**No time to address patients’ questions**			
	Set a collaborative agenda at the beginning of a visit	Ask the patient about their agenda, establish the number of concerns that can be addressed, and collaborate on a plan for any remaining issues.	Collaborative agenda-setting did not increase visit length, but reduced the amount of “oh by the way” concerns that surfaced at the end of the visit [51].
	Assess patients’ concerns and questions preemptively, prior to the visit	Assessing patients’ concerns could be completed either in paper-and-pencil format in a waiting room or via electronic check-in with artificial intelligence–powered chatbots and electronic visit planners.	Patients who used a visit planner in the waiting room were more prepared and began the visit by communicating their top concerns [52].
	Diversify modes of communication	Telehealth visits, e-visits, or communication via a patient portal could be helpful to meet patients’ remaining needs after clinical encounters.	Asynchronous e-visits through a patient portal provided clinical outcomes that were comparable to those provided by in-person care [53].
**No time to ensure patient understanding of clinical information**			
	Educational sessions	Use follow-up visits or education in small groups to clarify diagnosis and treatment options.	A group session saved 111 hours of clinical time, with 92% of patients increasing confidence regarding critical concepts in oncology care [54].
	Use educational materials about evidence-based treatment	Use standardized printed materials.	Patients who received printed chemotherapy information had almost 3 times greater odds of understanding their goals of care [18].
**No time to verify information that patients find on their own**			
	Direct patients to reliable digital resources	Preemptively advise patients on where to find high-quality resources for self-education, considering patients’ literacy, e-literacy, cultural background, and access to digital resources.	An example from a qualitative research, in which clinicians share their recommendations on how to help patients to stay informed: “...maybe suggest some more sites that will...have... more reputable information...direct them that way” [55].
	Contribute to increasing patients’ digital literacy	Share tips with patients on how to identify reliable information and direct patients to resources that might help them learn about finding reliable information, including educational materials and workshops.	The National Cancer Institute provides resources for patients on how to identify reliable information online [56].
	Engage other health care team members	Patients may address questions about information they find on their own to nurses, medical assistants, nutritionists, integrative specialists, physical therapists, and other experts in the health care system.	Nurse practitioners tend to adopt a patient-centered communication style while communicating about patients’ online health information–seeking [57].

One approach is to set a collaborative agenda at the start of the visit, allowing patients to identify their top concerns and helping HCPs prioritize what can be addressed while planning follow-up for unresolved issues. Previsit assessments, via paper forms or electronic tools such as check-in platforms and visit planners, can further streamline care. Artificial intelligence–powered chatbots may assist patients with lower digital literacy by helping them formulate and prioritize questions to prepare for clinical conversations. If visit time remains insufficient, we would like to underscore practices that many clinics currently strive to implement, such as follow-up through video, phone, or secure messaging. Additionally, to improve patient understanding of diagnoses and treatment options, structured educational sessions outside the primary visit, such as follow-up appointments or discussions in small groups, can provide deeper engagement. Further, offering standardized, evidence-based printed materials reinforces key clinical information and gives patients accessible, reliable resources to review on their own. In Table 1, you will find examples of how these strategies were implemented by clinical teams in different clinical contexts.

Recognizing that some patients will continue to seek information online, clinicians and support staff can preemptively guide patients to trustworthy sources that match their literacy levels, cultural backgrounds, and access to technology. Sharing strategies for evaluating information and offering digital literacy resources, such as educational tools or workshops, can further support this process. Finally, encouraging patients to consult other team members, such as nurses, medical assistants, nutritionists, or physical therapists, helps distribute the time needed for validating patient-identified information and ensures timely, accurate guidance.

While we focused this review on the US health care system, the challenge resonates globally, with multiple studies from other regions reporting issues related to insufficient clinical time between patients and HCPs [58-60]. In today’s landscape of highly complex and accessible public health information, which can be both helpful and potentially misleading, it is critical to test and implement strategies that counter time-limited oncology visits and help preserve patient trust while supporting their informed decision-making.

## Abbreviations

HCP: health care provider
